# A hybrid boundary element-finite element approach for solving the EEG forward problem in brain modeling

**DOI:** 10.3389/fnsys.2024.1327674

**Published:** 2024-05-03

**Authors:** Nasireh Dayarian, Ali Khadem

**Affiliations:** Department of Biomedical Engineering, Faculty of Electrical Engineering, K. N. Toosi University of Technology, Tehran, Iran

**Keywords:** EEG forward problem, finite element method, boundary element method, hybrid BE-FE method, MRI-based realistic head model

## Abstract

This article introduces a hybrid BE-FE method for solving the EEG forward problem, leveraging the strengths of both the Boundary Element Method (BEM) and Finite Element Method (FEM). FEM accurately models complex and anisotropic tissue properties for realistic head geometries, while BEM excels in handling isotropic tissue regions and dipolar sources efficiently. The proposed hybrid method divides regions into homogeneous boundary element (BE) regions that include sources and heterogeneous anisotropic finite element (FE) regions. So, BEM models the brain, including dipole sources, and FEM models other head layers. Validation includes inhomogeneous isotropic/anisotropic three- and four-layer spherical head models, and a four-layer MRI-based realistic head model. Results for six dipole eccentricities and two orientations are computed using BEM, FEM, and hybrid BE-FE method. Statistical analysis, comparing error criteria of RDM and MAG, reveals notable improvements using the hybrid FE-BE method. In the spherical head model, the hybrid BE-FE method compared with FEM demonstrates enhancements of at least 1.05 and 38.31% in RDM and MAG criteria, respectively. Notably, in the anisotropic four-layer head model, improvements reach a maximum of 88.3% for RDM and 93.27% for MAG over FEM. Moreover, in the anisotropic four-layer realistic head model, the proposed hybrid method exhibits 55.4% improvement in RDM and 89.3% improvement in MAG compared to FEM. These findings underscore the proposed method is a promising approach for solving the realistic EEG forward problems, advancing neuroimaging techniques and enhancing understanding of brain function.

## 1 Introduction

Electroencephalography (EEG) is a non-invasive, fast, and inexpensive method to record electrical potentials on the head surface. In neuroscience, it is important to characterize the sources of measured EEG signals and accurately localize them by solving an EEG inverse problem. The EEG inverse problem includes an EEG forward problem using a chosen source model (Grech et al., 2008; Güllmar et al., 2010; de Munck et al., 2017). The solution of the EEG forward problem yields an accurate calculation of the electromagnetic fields. To solve the EEG forward problem, the conductivity profile of the head is modeled, and the relation between the source model and the computed EEG signals is introduced in the EEG lead-field matrix, which can then be used to solve the EEG inverse problem. Then, the source locations and strengths are estimated from the measured EEG signals with the help of the EEG lead-field matrix obtained in the EEG forward model (Hallez et al., 2007; Akalin Acar and Makeig, 2013; de Munck et al., 2017; Vorwerk et al., 2017). Consequently, appropriate modeling of the EEG forward problem is an essential prerequisite for the accurate solution of the EEG inverse problem (Güllmar et al., 2010; Akalin Acar and Makeig, 2013). In other words, the physics of the problem is in the forward model, and errors caused by an inaccurate forward model cannot be corrected while solving the inverse problem.

Two advanced numerical methods called Finite Element Method (FEM) (de Munck et al., 2017; Vorwerk et al., 2017) and Boundary Element Method (BEM) (Stenroos and Sarvas, 2012; de Munck et al., 2017; Rahmouni et al., 2017) are widely used to solve the EEG forward problem. In the FEM, the entire volume is discretized into small elements (tetrahedral elements), and the potentials of all nodes are calculated. The FEM can easily incorporate arbitrary geometries and heterogeneous and anisotropic electrical conductivity of the head tissues (Wolters et al., 2006; Zhang et al., 2014; Beltrachini, 2019a). Unfortunately, the difficulty thorough using FEM is that it causes singularity when using the point dipoles as current dipoles in the EEG forward model, which increases the error of forward solution. However, the current dipoles are widely accepted models for modeling neuronal activities (Zhang et al., 2014; de Munck et al., 2017; Beltrachini, 2019a).

Some methods have been proposed to improve the behavior of the FEM in singularity cases, e.g., the subtraction method (Awada et al., 1997; Zhang et al., 2014; Beltrachini, 2019a; Darbas and Lohrengel, 2019) and the direct methods (Yan et al., 1991; Buchner et al., 1997; Hallez et al., 2007; Zhang et al., 2014). The subtraction method has a reasonable mathematical basis for point current dipole models. However, it is computationally relatively expensive and sensitive to conductivity jumps if the source is near them (Awada et al., 1997; Wolters et al., 2007; Lew et al., 2009; Beltrachini, 2019a,b). On the other hand, The direct FEM approaches such as St. Venant (Buchner et al., 1997) and partial integration (Yan et al., 1991) are easy to implement and have a much lower computational complexity, so they are very fast (Lew et al., 2009; Vorwerk et al., 2019b). However, the potential distribution strongly depends on the shape of the element at the source location (Awada et al., 1997; Medani et al., 2015). Among the direct approaches, the St. Venant approach was shown to have the most accurate results for the sources of not very high eccentricity (Vorwerk et al., 2019b). On the other hand the partial integration approach was shown to have higher stability even at the sources of high eccentricity (Medani et al., 2015).

On the other hand, the BEM is used for calculating the potentials of surface elements on the interface between compartments generated by a current source in piece-wise homogenous volume. The BEM can construct realistic geometry of piece-wise homogeneous isotropic compartments and solve the EEG forward problem accurately (de Munck et al., 2017). Also, it has numerical stability and effectiveness compared with differential equation-based techniques (Rahmouni et al., 2017; Monin et al., 2020). Unfortunately, the BEM formulations can be complicated to model complex geometry such as inhomogeneity, anisotropicity and surfaces with holes (Adde et al., 2003; de Munck et al., 2017; Rahmouni et al., 2017). Also, the BEM produces dense matrices that cause high computational cost compared with FEM (Adde et al., 2003).

In order to benefit from the advantages of both BEM and FEM, some coupled boundary element (BE)–finite element (FE) methods have been proposed in electromagnetic and biomedical problems (Bradley et al., 2001; Sikora et al., 2004; Olivi et al., 2010; Srinivasan et al., 2010; Ghaderi Daneshmand and Jafari, 2013). In Bradley et al. (2001), a new high-order cubic Hermite coupled FE/BE method has been proposed only for an isotropic three-layer spherical and realistic head model, and generalized Laplace’s equation had been solved. Also, a hybrid BE–FE method has been applied to the 2D forward problem of electrical impedance tomography (EIT) (Sikora et al., 2004) and has been used for modeling Diffusion equations in 3D multi-modality optical imaging (Srinivasan et al., 2010). A 3D coupling formulation was presented in Olivi et al. (2010) for solving the EEG forward problem iteratively. A domain decomposition (DD) framework was used to split the global system into several subsystems with smaller computational domains. Then, for each subsystem, one of the methods (BEM or FEM) was used. Several iterations were needed to solve the forward problem on the global system, and a relaxation parameter at each interface was compulsory to ensure convergence. The relaxation parameters were set manually, and an inappropriate value of these parameters would make the scheme diverge. Furthermore, three-layer concentric sphere models considering both the isotropic and anisotropic conductivity of the skull layer with dipoles of six locations and three orientations were modeled. No realistic head model was investigated. The coupling process was very time-consuming because the BEM and FEM ran iteratively until the relative residuals reached below a properly set value (6 × 10^−5^).

In Ghaderi Daneshmand and Jafari (2013), a hybrid BE–FE method, which directly combines the two BE and FE methods, has been proposed to solve the forward problem of EIT for a 3D cylindrical model of the human thorax. It should be noted that the EEG forward problem is completely different from the EIT forward problem regarding equations and boundary conditions. Thus, we must reformulate equations and extend them to be suitable for applying to the EEG forward problem. The advantage of using such a hybrid BE-FE method for solving the EEG forward problem is that the isotropic and homogeneous subregions containing the current dipoles can be modeled by the BEM and the other subregions (the inhomogeneous or anisotropic subregions or those without current dipoles) can be modeled by the FEM. Also, this method solves the global system in one step without any iteration. Consequently, it is expected that the application of the hybrid BE–FE method increases the accuracy of the EEG forward solution and consequently helps to more accurate localization of brain sources.

In this paper, BEM, partial integration FEM (PI-FEM), and hybrid BE–FE method, are employed to solve the EEG forward problem. To validate the hybrid BE-FE method in solving the EEG forward problem of isotropic multi-compartment media, we will use an isotropic piece-wise homogeneous three-layer spherical head model (brain, skull and scalp), which has an analytical forward solution, and the results will be compared with those of BEM and PI-FEM. To validate the hybrid BE-FE method in solving the EEG forward problem of anisotropic multi-compartment media, we will use an anisotropic three-layer spherical head model (brain, skull and scalp) in which the conductivity of the skull will be considered anisotropic and compare the results with those of PI-FEM. Since the cerebrospinal fluid (CSF) layer highly affects the scalp potentials (Ramon et al., 2006; Vorwerk et al., 2014), we will also investigate the performance of the hybrid BE-FE method compared with PI-FEM when considering the fourth layer for CSF and the anisotropic conductivity of the skull.

Because the conductivity uncertainties of head layers (especially skull and brain) have a significant influence on the EEG forward solution (Vorwerk et al., 2019a), we will repeat the simulation of each spherical head model 50 times with different realizations of conductivity of layers followed by statistical analysis to demonstrate the effect of conductivity uncertainties on the EEG forward solution. Finally, the hybrid BE-FE method and PI-FEM will be compared on a four-layer realistic head model in which the conductivity of the skull will be anisotropic.

This paper is organized as follows: In Section 2, the mathematical model for the EEG forward problem and its numerical solutions using BEM, PI-FEM, and hybrid BE–FE method was formulated. In Section 3, the performance criteria for validation were described. In Section 4, the results of simulated spherical and realistic head models were reported. In Section 5, the results were discussed. Finally, in Section 6, the paper was concluded, and some future works was proposed.

## 2 Mathematical model

### 2.1 EEG forward problem

**Figure 1:**
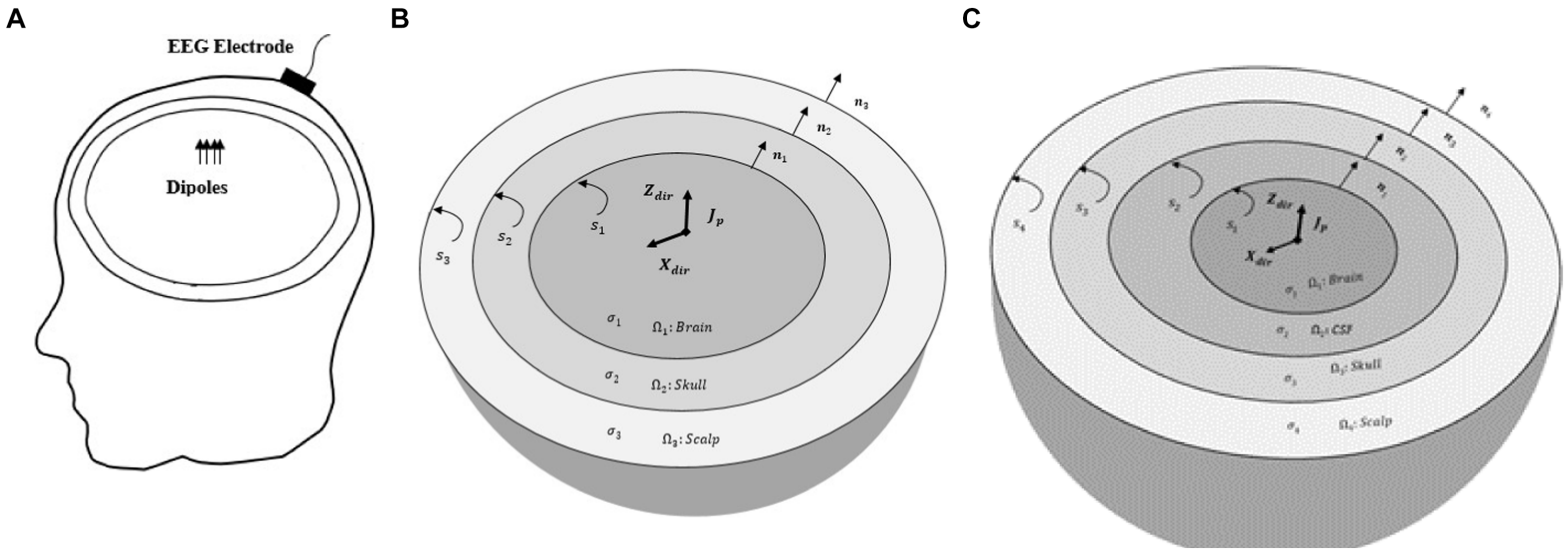
**(A)** EEG forward model. Electric potentials recorded by EEG electrodes are generated by dipoles within the head volume. A sample EEG electrode placed on the scalp is only shown. **(B)** The piece-wise homogenous three-layer spherical head model (brain, skull, and scalp). S1, S2, and S3 are the interfaces between brain-skull, skull-scalp, and scalp-air, respectively. Also, n1, n2 and n3 are the outward unit normal vectors of brain, skull and scalp layers. **(C)** The piece-wise homogenous four-layer spherical head model (brain, CSF, skull, and scalp). The conductivity of the skull can be isotropic or anisotropic. S1, S2, S3, and S4 are the interfaces between brain-CSF, CSF-skull, skull-scalp, and scalp-air, respectively. n1, n2, n3, and n4 are the outward unit normal vectors of brain, CSF, skull and scalp layers, respectively.

The EEG forward problem entails calculating the electric potential 𝜑 on the scalp surface 𝑆 (see Figure 1A). These potentials are generated by current dipoles within the head volume R. Therefore, since the relevant frequencies of the EEG spectrum are below 100 Hz, the quasi-static approximation of Maxwell’s equations is used to estimate the electric potentials over the scalp with homogeneous Neumann boundary conditions at each time sample (t) as follows (Hämäläinen et al., 1993):


(1) $$\begin{array}{c} \nabla .\left(\sigma \nabla \varphi \left(t\right)\right)=\nabla .J_{P}\left(t\right)\;\mathrm{inside}\;R\;\mathrm{for each time sample}\;\left(t\right)\\ \sigma \;\partial \varphi /\partial n=0\;\mathrm{on}\;S \end{array}$$

where 𝜎: ℝ^3^ → ℝ^3^ denotes conductivity tensor of tissue conductivity in R and 𝑱_𝑷_ denotes the primary current density of the brain source. Also, ***n*** is the outward unit normal vector at the surface *S (*Hämäläinen et al., *1993*; de Munck et al., *2017**)*. In this manuscript, vector quantities are denoted by bold characters.

The primary current density 𝑱_𝑷_ is commonly modeled as two delta functions at the current source position

𝒓_2_ (𝑥_2_, 𝑦_2_, 𝑧_2_) and the current sink position 𝒓_𝟏_ (𝑥_1_, 𝑦_1_, 𝑧_1_) with the current source density *I* as follows (Hallez et al., 2007):


(2) $$\nabla \cdot J_{P}\left(x\right)=I\left[\delta \left(r–r_{2}\right)-\delta \left(r–r_{1}\right)\right]$$

### 2.2 Boundary element method

In the Boundary element method (BEM), the reciprocal relation is applied to derive a boundary integral equation for the boundary value problem Equation (1) as given by Ang (2007).


(3) $$\begin{array}{c} \lambda \left(\xi ,\eta ,\zeta \right)\varphi \left(\xi ,\eta ,\zeta \right)-\underset{\underset{R}{∭}\Phi_{3D}\left(x,y,z;\xi ,\eta ,\zeta \right)g\left(x,y,z\right)\mathrm{dxdydz}}{\;}\\ \;=\underset{S}{\iint }[\varphi \left(x,y,z\right)\frac{\partial }{\partial n}\;\left(\Phi_{3D}\left(x,y,z;\xi ,\eta ,\zeta \right)\right)\\ \;-\Phi_{3D}\left(x,y,z;\xi ,\eta ,\zeta \right)\frac{\partial }{\partial n}\;\left(\varphi \left(x,y,z\right)\right)]\;ds\left(x,y,z\right) \end{array}$$

where 𝑔 (𝑥, 𝑦, 𝑧) = 1/𝜎∇. 𝑱_*P*_, λ(𝜉, 𝜂, 𝜁) is a characteristic function of the domain *R*, 𝜎 is the conductivity of domain that must be constant and isotropic. The function 𝛷_3D_ (𝑥, 𝑦, 𝑧; 𝜉, 𝜂, 𝜁) is the fundamental solution of the three dimensional Laplace’s equation and is given by Equation (4) Ang (2007).


(4) $$\Phi_{3D}\left(x,y,z;\xi ,\eta ,\zeta \right)=-\frac{1}{4\pi \sqrt{{\left(x-\xi \right)}^{2}+{\left(y-\eta \right)}^{2}+{\left(z-\zeta \right)}^{2}}}$$

Consider the surface of a region to be discretized to *N* triangular elements. The potential ϕ (𝑥, 𝑦, 𝑧) and its normal derivative 𝜕/𝜕𝑛 [𝜑(𝑥, 𝑦, 𝑧)] are approximated as constant values over each element as Equation (5):


(5) $$\varphi ≃{\bar{\varphi }}^{\left(k\right)}\;\mathrm{and}\;\frac{\partial \varphi }{\partial n}≃{\bar{p}}^{\left(k\right)}\;for\;\left(x,y,z\right)\in S^{\left(k\right)}\;\left(k=1,2,\ldots ,N\right)$$

where 𝜑̅^(𝑘)^and 𝑝̅^(𝑘)^ are the average values of 𝜑 and 𝜕ϕ/𝜕𝑛 on the centroid point of the k^th^ surface element, 𝑆^(𝑘)^.

Using these approximations, Equation (3) is simplified as


(6) $$\begin{array}{c} \lambda \left(\xi ,\eta ,\zeta \right)\varphi \left(\xi ,\eta ,\zeta \right)-\underset{\underset{R}{∭}\Phi_{\;3D}\left(x,y,z;\xi ,\eta ,\zeta \right)g\left(x,y,z\right)\mathrm{dxdydz}}{\;}\;\\ =\overset{N}{\underset{k=1}{\sum }}\left\{{\bar{\varphi }}^{\left(k\right)}D_{2}^{\left(k\right)}\left(\xi ,\eta ,\zeta \right)-\bar{p}\left(k\right)D_{1}^{\left(k\right)}\left(\xi ,\eta ,\zeta \right)\right\} \end{array}$$

where


(7) $$\begin{array}{c} D_{1}^{\left(k\right)}\;\;\left(\xi ,\eta ,\zeta \right)=\underset{\iint_{S^{\left(k\right)}}\Phi_{3D}\left(x,y,z;\xi ,\eta ,\zeta \right)ds\left(x,y,z\right)}{\;}\\ D_{2}^{\left(k\right)}\left(\xi ,\eta ,\zeta \right)=\underset{\iint_{S^{\left(k\right)}}\frac{\partial }{\partial n}\left(\Phi_{3D}\left(x,y,z;\xi ,\eta ,\zeta \right)\right)ds\left(x,y,z\right)\;}{\;}\;\; \end{array}$$

Let (𝜉, 𝜂, 𝜁) in Equation (6) be given consecutively by the centroid point of *S*^(1)^, *S*^(2)^, … , *S*^(N)^. D_1_^(*k*)^ and D_2_^(*k*)^ were introduced in Equation (7). Consequently, Equation (6) can be rewritten as Ang (2007).


(8) $$\begin{array}{l} \frac{1}{2}{\bar{\varphi }}^{\left(m\right)}-\underset{\underset{R}{∭}\Phi_{3D}\left(x,y,z;\xi ,\eta ,\zeta \right)g\left(x,y,z\right)\mathrm{dxdydz}}{\;}\\ =\overset{N}{\underset{k=1}{\sum }}\left\{{\bar{\varphi }}^{\left(k\right)}D_{2}^{\left(k\right)}\left({\bar{x\;}}^{\left(m\right)},{\bar{y\;}}^{\left(m\right)},{\bar{z\;}}^{\left(m\right)}\right)-{\bar{p\;}}^{\left(k\right)}D_{1}^{\left(k\right)}\left({\bar{x\;}}^{\left(m\right)},{\bar{y\;}}^{\left(m\right)},{\bar{z\;}}^{\left(m\right)}\right)\right\}\;\\ for\;m=1,2,\ldots ,N \end{array}$$

where (𝑥̅^(𝑚)^, 𝑦̅^(𝑚)^, 𝑧̅^(𝑚)^) is the centroid point of the element 𝑆^(𝑚)^. On the typical element *S*^(𝑘)^, either 𝜑̅^(𝑘)^ or 𝑝̅^(𝑘)^ is known. Thus Equation (8) constitutes a system of *N* linear algebraic equations including *N* unknowns for a one-layer homogenous region as given by Ang (2007).


(9) $$A_{\mathrm{BE}}\;X_{\mathrm{BE}}=B_{\mathrm{BE}}$$

where 𝑋_𝐵𝐸_ is the column vector (column matrix) including both 𝜑̅ and 𝑝̅ for each element, 𝐴_𝐵𝐸_ is the coefficient matrix and 𝐵_𝐵𝐸_ is the column vector containing values of boundary condition and the current dipoles information. Equation (9) describes the matrix form of the BEM for just one homogenous subregion.

In order to implement BEM for multi-layer piece-wise homogeneous media, we used the same approach as that of Ghaderi Daneshmand and Jafari (2013). Hereby, we describe that method for a three-layer spherical head model with nested regions of constant conductivity (see Figure 1B). First, all interfaces are discretized to *N* triangles, and then the first subregion (brain) is represented by Equation (1). Other subregions (skull and scalp) are represented by the Laplace equation. The first and second boundaries of each subregion are assumed to be Dirichlet and Neumann conditions, respectively. Considering the air/scalp interface, the boundary condition of the second boundary of the scalp is of Neumann type. The system of linear algebraic equations for each subregion is obtained as Equation (10) (Ang, 2007):


(10) $$\begin{array}{cc} A_{{\mathrm{BE}}_{j}}X_{{\mathrm{BE}}_{j}}=B_{{\mathrm{BE}}_{j}} & \;j=1,2,3 \end{array}$$

where 𝑋_𝐵𝐸j_ is the column vector including both 𝜑̅ and 𝑝̅ for each element at the *j*^th^ subregion. Assembling the

equations corresponding to all subregions, the same algebraic equation as Equation (9) is derived. Then, continuity conditions at the interface between two adjacent subregions should be applied to the resulting equation. The continuity condition for the electric potential and continuity condition for the normal component of the current density at the interface surface 𝑆_𝐵𝐸−𝐵𝐸_ are given as Equation (11) (Ang, 2007):


(11) $$\begin{array}{c} \varphi_{{\mathrm{BE}}_{i}}=\varphi_{{\mathrm{BE}}_{j}}\\ \sigma_{{\mathrm{BE}}_{i}}\frac{\partial \varphi_{BE_{i}}}{\partial n_{i}}=\sigma_{BE_{j}}\frac{\partial \varphi_{BE_{j}}}{\partial n_{j}} \end{array}$$

where (𝜑_𝐵𝐸𝑖_, 𝜎_𝐵𝐸𝑖_) and (𝜑_𝐵𝐸𝑗_, 𝜎_𝐵𝐸𝑗_) are the (electric potential, conductivity) of each element on the interface between two adjacent subregions Ω_𝐵𝐸𝑖_ and Ω_𝐵𝐸𝑗_, respectively. After the continuity condition for the electric potential and the normal component of the current density, the electric potential and its normal derivation of surface elements are computed by using Gaussian elimination method.

### 2.3 Finite element method using partial integration approach (PI FEM)

In the FEM, the functional *F*(𝜑) derived from the Rayleigh-Ritz method, a variational method, is minimized to solve the boundary value problems. Equation (1) can be written in Cartesian coordinates with homogeneous Neumann boundary conditions Equation (12) (Jin, 2014).


(12) $$\begin{array}{c} \frac{\partial }{\partial x}\left(\sigma_{x}\frac{\partial \varphi }{\partial_{x}}\right)+\frac{\partial }{\partial_{x}}\left(\sigma_{y}\frac{\partial \varphi }{\partial_{y}}\right)+\frac{\partial }{\partial_{z}}\left(\sigma_{z}\frac{\partial \varphi }{\partial_{z}}\right)=\nabla \cdot J_{P}\\ \sigma \frac{\partial \varphi }{\partial n}=0\;\mathrm{on}\;S \end{array}$$

Consequently, the functional is defined as Jin (2014):


(13) $$\begin{array}{l} F\left(\varphi \right)=\frac{1}{2}\;\underset{R}{\int }\left[\sigma_{x}{\left(\frac{\partial \varphi }{\partial x}\right)}^{2}+\sigma_{y}{\left(\frac{\partial \varphi }{\partial y}\right)}^{2}+\sigma_{z}{\left(\frac{\partial \varphi }{\partial z}\right)}^{2}\right]dR\\ \;-\underset{R}{\int }\nabla \cdot J_{P}\varphi dR \end{array}$$

where 𝜎_𝑥_, 𝜎_𝑦_ and 𝜎_𝑧_ are, respectively, the conductivity along x, y and z axes that are constant and equal to each other in isotropic media but unequal to each other in anisotropic media.

The first step of the FEM is the discretization of the regions into a number of tetrahedral elements. The unknown potential 𝜑^𝑒^ at any point within each tetrahedral element can be approximated as Equation (14) Jin (2014):


(14) $$\varphi^{e}\left(x,y,z\right)=\overset{4}{\underset{j=1}{\sum }}N_{j}^{e}\left(x,y,z\right)\varphi_{j}^{e}$$

where the interpolation functions 𝑁_j_^𝑒^(𝑥, 𝑦, 𝑧) are given by Equation (15).


(15) $$N_{j}^{e}\left(x,y,z\right)=\frac{1}{6V^{e}}\left(a_{j}^{e}+b_{j}^{e}x+c_{j}^{e}y+d_{j}^{e}z\right)$$

where 𝑎_*j*_^𝑒^, 𝑏_*j*_^𝑒^, 𝑐_*j*_^𝑒^ and 𝑑_*j*_^𝑒^ are constants and are determined from the coordinates of the nodes of elements (Jin, 2014). By minimizing the functional 𝐹(𝜑) in Equation (13) for each element and assembling all elements in the whole volume, and using the partial integration (PI) approach to model the current dipoles (Yan et al., 1991; Schimpf et al., 2002), the final set of equations can be written as follows (Jin, 2014).


(16) $$K_{FE}\;\varphi_{FE}=B_{FE}$$

where 𝐾_𝐹𝐸_ is the coefficient (stiffness) matrix, which is a function of nodal coordinates and conductivity of each element. 𝜑_𝐹𝐸_ is the column vector of the unknown electric potential of the nodes and 𝐵_𝐹𝐸_ is the source column vector contributed by the dipoles that has non-zero entries for the set of nodes of the elements that contain the dipoles. After the Neumann boundary condition, given in Equation (1) is applied to Equation (16), the electric potential is computed by using quasi-minimal residual method. It is noteworthy that in FEM, another approach called Galerkin which is the weighted residuals method can be used (Jin, 2014; Munafò et al., 2023).

### 2.4 Hybrid BE–FE method

The hybrid BE–FE formulation consists of both FE and BE formulations. It is implemented by combining Equation (9) with Equation (16) and applying boundary conditions in each interface. First, the BEM is used to represent the Poisson Equation (1) at the brain subregion (BE region) considering the Dirichlet boundary condition. Then FEM is used to represent the Laplace equation (∇. (𝜎∇𝜑) = 0) at other subregions (FE regions) considering Neumann condition at skull/brain and air/scalp interfaces for the three-layer spherical head model, and CSF/brain and air/scalp interfaces for the four-layer spherical head model to derive Equation (16). The values of boundary conditions on each interface are unknown. Assembling Equations (9) and (16) gives.


(17) $$\left[\begin{array}{cc} K_{FE} & 0\\ 0 & A_{\mathrm{BE}} \end{array}\right]\left[\begin{array}{c} \varphi_{\mathrm{BE}}\\ X_{\mathrm{BE}} \end{array}\right]=\left[\begin{array}{c} B_{FE}\\ B_{\mathrm{BE}} \end{array}\right]$$

To solve Equation (17), the value of boundary condition at the BE-FE interface must be applied but it is unknown. To apply boundary condition on a BE-FE interface S_𝐵𝐸−𝐹𝐸_, the potential on S_𝐵𝐸−𝐹𝐸_ computed from both BE region and FE region must be the same. Since the potential on a surface BE element is constant, but in a FE element is linear, in order to equalize those potentials on the BE-FE interface, one may take the average of three FE nodal potentials and obtain the following approximation for the BE surface element potential as Ghaderi Daneshmand and Jafari (2013).


(18) $$\varphi_{\mathrm{BE}}=\frac{\varphi_{FE_{1}}+\varphi_{FE_{2}}+\varphi_{FE_{3}}}{3}$$

where 𝜑_𝐹𝐸1_, 𝜑_𝐹𝐸2_, and 𝜑_𝐹𝐸3_ are FE nodal potentials at each element in S_𝐵𝐸−𝐹𝐸_. The continuity condition for the normal component of the current density at S_𝐵𝐸−𝐹𝐸_ yields Equation (19) (Ghaderi Daneshmand and Jafari, 2013).


(19) $$\sigma_{FE}\frac{\partial \varphi_{FE}}{\partial n_{FE}}=-\sigma_{\mathrm{BE}}\frac{\partial \varphi_{\mathrm{BE}}}{\partial n_{\mathrm{BE}}}$$

Applying Equations (18, 19) to (17), the matrices 𝐾_𝐹𝐸_, 𝐴_𝐵𝐸_, 𝐵_𝐹𝐸_, and 𝐵_𝐵𝐸_, are modified as 𝐾̃_𝐹𝐸_, 𝐴̃_𝐵𝐸_, 𝐵̃_𝐹𝐸_, and 𝐵̃_𝐵𝐸_, respectively; and the resulting equation is obtained as.


(20) $$\left[\begin{array}{cc} {\tilde{K}}_{FE} & M_{FE}\\ M_{\mathrm{BE}} & {\tilde{A}}_{\mathrm{BE}} \end{array}\right]\left[\begin{array}{c} \varphi_{FE}\\ X_{\mathrm{BE}} \end{array}\right]=\left[\begin{array}{c} {\tilde{B}}_{FE}\\ {\tilde{B}}_{\mathrm{BE}} \end{array}\right]$$

where 𝑀_𝐵𝐸_ and 𝑀_𝐹𝐸_ are sparse matrices constructed as a result of applying Equations (18, 19), respectively. Using Gaussian elimination method, Equation (20) is solved for 𝜑_𝐹𝐸_ and 𝑋_𝐵𝐸_.

To use the hybrid BE-FE method, two important points should be considered:

First, when an anisotropic piece-wise homogenous multi-layer medium is modeled, the layer(s) containing dipoles must be modeled with the BEM, and the layers with anisotropic conductivity must be modeled with the FEM. Other layers can be modeled with each of FEM or BEM depending on our goals, such as less time and memory consumption, and computational complexity. So in this paper, we used the FEM for other layers. Also, we will show the performance of the hybrid BE-FE method when only one layer, including current dipoles is modeled with the BEM, and we compare this performance with the performance of FEM.

Second, we used linear elements in the FEM. Thus, to couple BEM and FEM elements, we need constant triangular elements or continuous linear triangular elements in BE domain to use Equations (18, 19). We prefer to use constant elements in the BEM because solving forward problem with constant elements is more accurate than continuous linear elements (Ang, 2007).

It should be noted though the discontinuous linear element is more accurate than constant and continuous linear elements, it cannot be used to couple with the FEM in our method and because in our method, we need the constant potential in the surface BE elements or the vertex’s potentials of BE elements to use in Equation (18).

### 2.5 Tetrahedral mesh generation

To implement the FEM and BEM, the entire region is first discretized into tetrahedral volume elements for the FEM, and then the required surface triangular meshes in the BEM are prepared from the data of tetrahedral elements of the entire region. For mesh extraction, we use ISO2MESH (Fang and Boas, 2009) that provides us with accurate mesh volume and surface elements.

In the hybrid BE–FE method, the BE regions and the FE regions are discretized using a triangular surface mesh and a tetrahedral volume mesh, respectively. In order that the BE and FE regions in the hybrid method have the same boundary surface elements, the entire volume domain is first discretized by irregular tetrahedral volume elements, and then irregular triangular surface elements for the BE regions are extracted from the data of the tetrahedral elements. It is noteworthy that the FEM uses the entire tetrahedral volume elements.

In the realistic head model, we can leverage on FieldTrip software to obtain an automatic segmentation of the brain, CSF, skull, and scalp (Oostenveld et al., 2011). By using FieldTrip, the surface boundaries of these four structures are extracted. Then, based on the boundaries, a finite element model can be constructed by ISO2MESH (Fang and Boas, 2009).

## 3 Validation method

### 3.1 Validation platform

To validate the precision of the proposed hybrid BE-FE method, it was essential to employ a model with a known analytical solution within the domain of bioelectromagnetism, serving as a reliable reference or “ground truth.”

To fulfill this requirement, we opted for spherical models due to their similarity to real-world scenarios and the availability of analytical solutions. So, the proposed hybrid BE-FE method is validated and compared with the FEM and BEM, with simulating piece-wise homogeneous three- and four-layer spherical head models.

We will first simulate an isotropic, piece-wise homogeneous three-layer spherical head model with radial and tangential dipoles of six different eccentricities. Then, we will repeat the same simulation but with an anisotropic layer (skull) to show the performance of FEM and the proposed hybrid BE-FE method when the skull is modeled as a layer of homogenous and anisotropic profile. Afterward, we will add a fourth layer for CSF to the previous anisotropic model. This layer has an important role in distributing the currents in the head model and scalp potentials (Ramon et al., 2006; Vorwerk et al., 2014). Next, we will repeat the same procedure to assess the performance of the hybrid BE-FE method on this anisotropic and piece-wise homogeneous four-layer medium.

To extend the applicability of our findings to authentic scenarios, we will conduct additional investigations using a real head model. Consequently, we will compare the proposed hybrid BE-FE method with the FEM on an anisotropic piece-wise homogeneous four-layer realistic head model. Based on this comprehensive approach, encompassing both analytical and real-world evaluations, will contribute to a robust validation of the efficacy and reliability of our proposed numerical method.

To assess the precision of our proposed numerical method in addressing the forward problem and its associated influencing factors, it is essential to hold all influencing factors constant while varying only one selection variable. These factors are the precision of head model segmentation, mesh quality, conductivity variations in different head layers, and the positioning and orientation of dipoles (Miinalainen et al., 2019; Vorwerk et al., 2019a; Nielsen et al., 2022). Therefore, we will evaluate hybrid BE-FE method according to conductivity uncertainties of head layers for each of the above spherical head models and dipoles’ positions and orientations. We will simulate 50 realizations using randomly chosen conductivity values from realistic intervals. This allows us to gain a statistical overview of the precision of solving EEG forward problem with regard to the electromagnetic properties of head layers.

In each of the above models, unit radial (along z-axis) and tangential (along x-axis) dipoles of six different eccentricities will be considered. For each dipole, the source eccentricity is defined as the percentage of the Euclidian distance between the dipole location and the sphere center divided by the radius of the innermost shell (Wagner et al., 2016). In our implementations, the most eccentric dipole has an eccentricity of 98%.

In this paper, for spherical head models, we use the analytical solution given in Zhi (1995) that calculates the electric potential on the outmost surface (scalp) of isotropic/anisotropic multi-layer spherical head model generated by a dipole inside the innermost layer (brain). For the realistic head model, we use a refined model of the FEM to obtain a reference FE solution because an analytical solution is unavailable for real models.

It is noteworthy that in all of our simulations, in order to have fair comparisons between the accuracy of the FEM, BEM and hybrid BE-FE method, we choose the mesh resolution that their computation times are nearly the same.

### 3.2 Error criteria

The error between the numerical solution and analytical solution can be obtained by Relative Difference Measure (RDM) and the Magnitude ratio (MAG) (Mejis et al., 1989; de Munck et al., 2017). These measures are, respectively, defined as Equations (21) and (22):


(21) $$RDM=|\frac{\varphi_{\mathrm{Ana}}}{\left|\varphi_{\mathrm{Ana}}\right|}-\frac{\varphi_{Num}}{\left|\varphi_{Num}\right|}|$$

(22) $$MAG=|\frac{\varphi_{Num}}{\varphi_{\mathrm{Ana}}}|$$

where 𝜑_𝐴𝑛𝑎_ and 𝜑_𝑁𝑢𝑚_ are analytical and numerical solutions, respectively and | | denotes the square root of Euclidean distance. In this paper, 𝜑_𝐴𝑛𝑎_ is calculated by using the analytical solution given in Zhi (1995) for spherical head models and the reference FE solution obtained from the refined model of the FEM for the realistic head model.

## 4 Simulation results

In this paper, the performance of the hybrid BE-FE method to solve the EEG forward problem will be assessed using both spherical and realistic head models. In the spherical models, the solution will be evaluated on all outer boundary (scalp) nodes instead of the small number of them so that the results are nearly independent of the choice of electrode positions (Vorwerk et al., 2017). On the other hand, for the realistic head model, the results will be assessed on 90 positions of the scalp surface. It is noteworthy that other influential factors on solving forward problem were considered completely the same.

### 4.1 Example I: isotropic piece-wise homogenous three-layer spherical head model

To compare the performance of hybrid BE-FE method with the BEM and PI-FEM, a numerical validation will be performed using a three-compartment (brain, skull and scalp) spherical head model as shown in Figure 1B with parameters indicated in Table 1 (Malmivuo and Plonsey, 2002; Vorwerk et al., 2019a). The optimized anisotropy ratio in Table 1 is defined as the ratio of radial conductivity (𝜎_𝑟_) to tangential conductivity (𝜎_𝑡_) of the skull (Dannhauer et al., 2011). In this simulation, the PI-FEM mesh has 6,808 nodes and 34,428 tetrahedral volume elements, the BEM mesh has 3,092 triangular surface elements, and the hybrid BE-FE mesh has 2,578 nodes, 10,688 tetrahedral volume elements and 1894 triangular surface elements.

**Table 1:** Parameters of the concentric three-layer spherical head model (Malmivuo and Plonsey, 2002; Vorwerk et al., 2019a).

Tissue	Brain	Skull	Scalp
Outer shell radius (cm)	8.0	8.6	9.2
Conductivity interval (S/m)	0.2200–0.6700	0.0016–0.0330	0.2800–0.8700
Optimized anisotropy ratio (𝝈_𝒓_/𝝈_𝒕_)	-	0.0093: 0.015	-

The conductivity values, radius and optimized anisotropy ratio are based on Vorwerk et al. (2019a), Malmivuo and Plonsey (2002), and Dannhauer et al. (2011), respectively.

**Figure 2:**
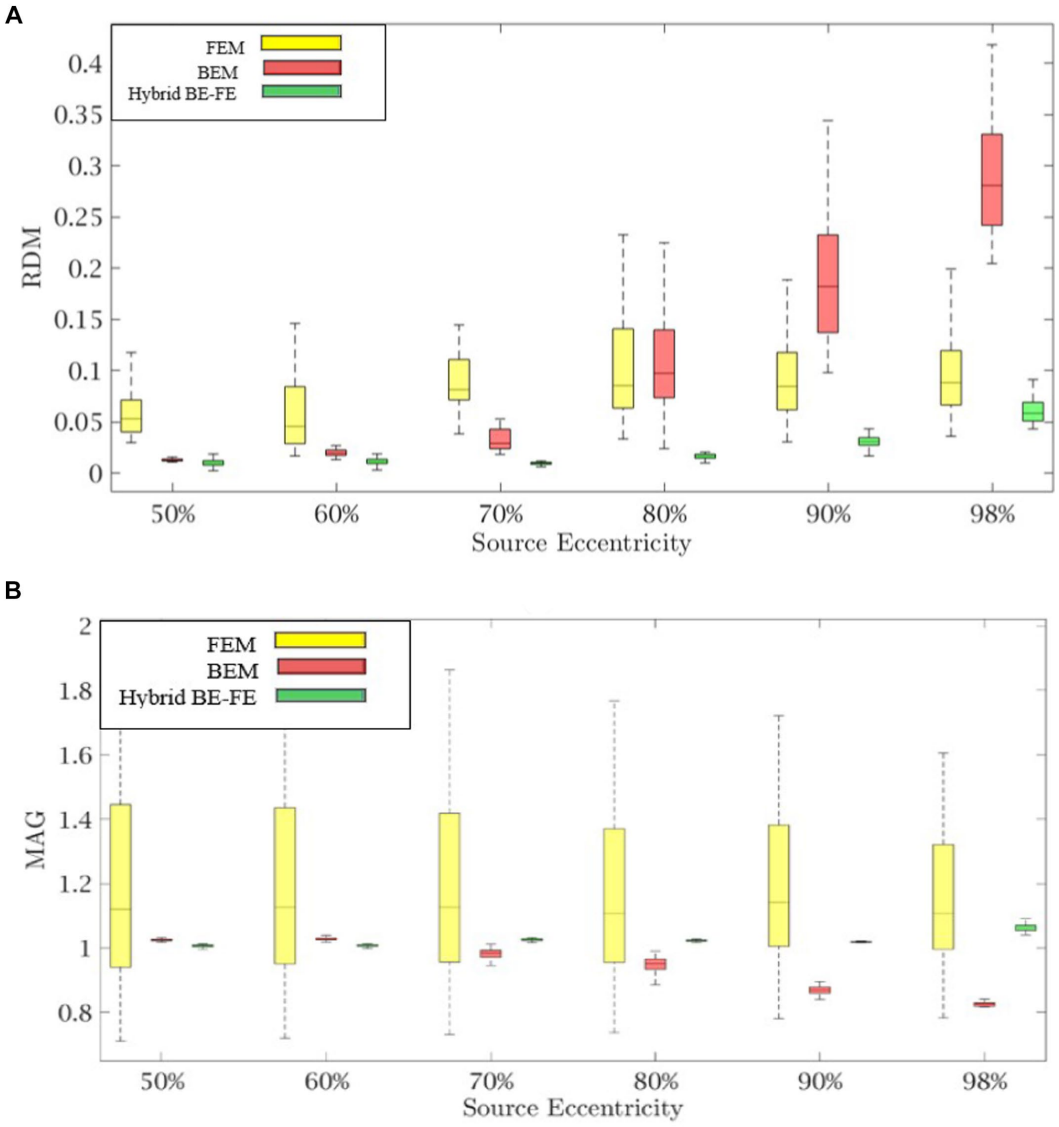
Example I: isotropic piece-wise homogenous three-layer spherical head model for radial dipole orientation (z-axis), **(A)** RDM and **(B)** MAG boxplots of PI-FEM, BEM, and hybrid BE-FE method at six different source eccentricities.

**Figure 3:**
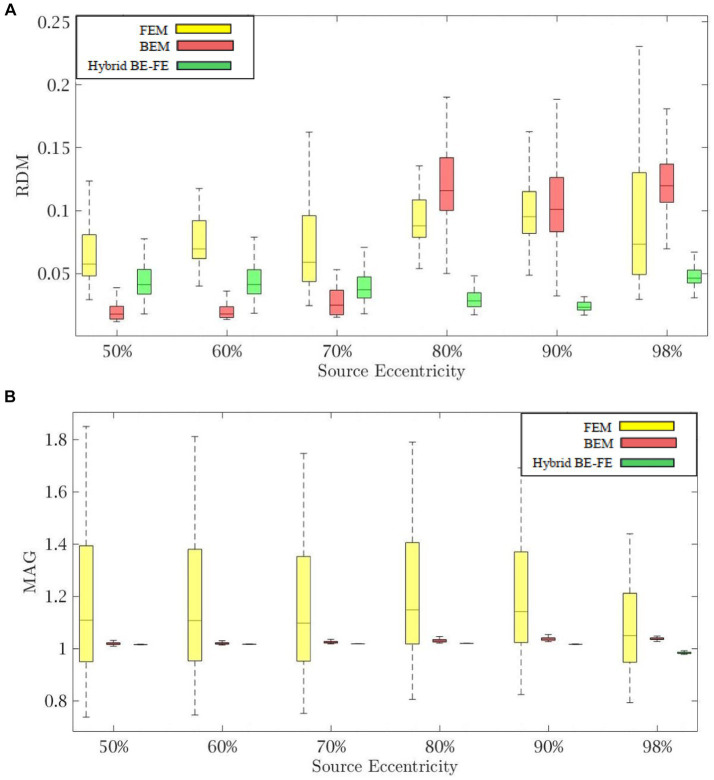
Example I: isotropic piece-wise homogenous three-layer spherical head model for tangential dipole orientations (x-axis), **(A)** RDM and **(B)** MAG boxplots of PI-FEM, BEM, and hybrid BE-FE method at six different source eccentricities.

Figures 2, 3 show boxplots of RDM and MAG of PI-FEM, BEM, and hybrid BE-FE method for isotropic and piece-wise homogeneous three-layer spherical head models versus different eccentricities of the dipole for radial and tangential dipoles, respectively. For each model, 50 realizations were simulated by randomly chosen conductivities from intervals shown in Table 1. Also, the mean and standard deviation of RDM and MAG and *p*-values of Wilcoxon signed-rank tests are reported in Table 2. It should be noted that some datasets did not pass the Gaussian test (*p*-value < 0.05). For this reason, we used Wilcoxon signed-rank test to calculate *p*-values. The significant differences are shown as gray in Table 2.

**Table 2:** Example I: isotropic piece-wise homogeneous three-layer spherical head model, mean ± std. and *p*-value of Wilcoxon signed-rank test of 50 realizations of RDM and MAG obtained from PI-FEM, BEM and hybrid BE-FE method for dipoles of six different source eccentricities and both radial and tangential directions.

		Radial direction (z-axis)						Tangential direction (x-axis)					
	Source eccentricity	50%	60%	70%	80%	90%	98%	50%	60%	70%	80%	90%	98%
RDM	PI- FEM	0.05981 ± 0.0253	0.05644 ± 0.0334	0.09152 ± 0.0313	0.101 ± 0.0492	0.0945 ± 0.039	0.0955 ± 0.0405	0.0637 ± 0.0221	0.0763 ± 0.0235	0.0699 ± 0.0338	0.097 ± 0.0284	0.1004 ± 0.0295	0.0902 ± 0.0508
	BEM	0.0128 ± 0.0011	0.0194 ± 0.0035	0.0379 ± 0.0242	0.1154 ± 0.0701	0.2119 ± 0.0907	0.2831 ± 0.0725	0.0197 ± 0.0073	0.0218 ± 0.0116	0.0301 ± 0.0169	0.1388 ± 0.0669	0.1277 ± 0.0762	0.1269 ± 0.0355
	Hybrid BE-FE	0.0100 ± 0.0035	0.0114 ± 0.0034	0.0107 ± 0.0044	0.0157 ± 0.0035	0.0301 ± 0.0069	0.0639 ± 0.0189	0.0463 ± 0.0206	0.0465 ± 0.0208	0.0419 ± 0.0185	0.0311 ± 0.0115	0.0245 ± 0.0054	0.049 ± 0.012
	*P*-value1	7.5569 × 10^−10^	7.5569 × 10^−10^	1.3238 × 10^−7^	0.4544	6.0236 × 10^−9^	9.6344 × 10^−10^	9.6344 × 10^−10^	1.3025 × 10^−9^	7.5248 × 10^−7^	0.00013	0.1167	0.0015
	*P*-value2	7.5569 × 10^−10^	7.5569 × 10^−10^	7.5569 × 10^−10^	7.5569 × 10^−10^	7.5569 × 10^−10^	1.7253 × 10^−6^	0.0021	1.0972 × 10^−5^	0.00017	8.0311 × 10^−10^	7.5569 × 10^−10^	2.9793 × 10^−5^
	*P*-value3	8.0203 × 10^−6^	1.6552 × 10^−9^	7.5569 × 10^−10^	7.5569 × 10^−10^	7.5569 × 10^−10^	1.0872 × 10^−9^	6.3812 × 10^−9^	1.0235 × 10^−9^	7.5569 × 10^−10^	7.5569 × 10^−10^	7.5569 × 10^−10^	7.5569 × 10^−10^
MAG	PI-FEM	1.1852 ± 0.2257	1.1856 ± 0.2515	1.1817 ± 0.2733	1.158 ± 0.2875	1.1856 ± 0.2946	1.1481 ± 0.1944	1.1609 ± 0.2631	1.1567 ± 0.2527	1.1431 ± 0.2377	1.1979 ± 0.2284	1.1845 ± 0.2038	1.0774 ± 0.1586
	BEM	1.0268 ± 0.006	1.02875 ± 0.0212	0.9795 ± 0.0212	0.9431 ± 0.0364	0.861 ± 0.0305	0.8178 ± 0.0273	1.0201 ± 0.0077	1.0222 ± 0.0085	1.0271 ± 0.0087	1.035 ± 0.0171	1.0423 ± 0.019	1.0393 ± 0.0077
	Hybrid BE-FE	1.0069 ± 0.0044	1.0082 ± 0.0046	1.0294 ± 0.0138	1.025 ± 0.0142	1.0198 ± 0.003	1.0709 ± 0.0298	1.0159 ± 0.0019	1.0172 ± 0.0018	1.0187 ± 0.0013	1.0203 ± 0.0006	1.0166 ± 0.0007	0.9836 ± 0.0034
	*P*-value1	0.0017	0.0015	8.3898 × 10^−6^	2.8864 × 10^−7^	1.0872 × 10^−9^	8.0311 × 10^−10^	0.0016	0.002	0.0053	4.348 × 10^−5^	5.5719 × 10^−5^	0.166
	*P*-value2	0.0004	0.0003	0.0014	0.0023	2.0268 × 10^−5^	0.0267	0.0014	0.0014	0.0023	7.3274 × 10^−6^	2.9108 × 10^−6^	0.0005
	*P*-value3	7.5569 × 10^−10^	7.5569 × 10^−10^	7.5569 × 10^−10^	7.5569 × 10^−10^	7.5569 × 10^−10^	7.5569 × 10^−10^	8.0203 × 10^−6^	3.3651 × 10^−7^	9.0681 × 10^−10^	8.0311 × 10^−10^	7.5569 × 10^−10^	7.5569 × 10^−10^

*p*-value1 corresponds to comparing the results of PI-FEM, and BEM, *p*-value2 corresponds to comparing the results of PI-FEM and hybrid BE-FE method, and *p*-value3 corresponds to comparing the results of BEM and hybrid BE-FE method. The significant results are shown as gray.

Comparing the PI-FEM, BEM, and hybrid BE-FE method with regard to the RDM for isotropic piece-wise homogeneous three-layer spherical head model (Figure 1A) shows that the hybrid BE-FE method significantly outperforms the PI-FEM for dipoles of both radial (Figure 2A) and tangential (Figure 3A) directions and all six eccentricities. In the radial direction, the hybrid BE–FE has a maximum RDM of 0.0639 ± 0.0189 at 98% source eccentricity, and the PI-FEM has its maximum RDM of 0.0955 ± 0.0405 at the same source eccentricity (Figure 2A). Also, the PI-FEM leads to a larger RDM variance than the hybrid BE-FE method. In the tangential direction, the maximum RDM obtained from the hybrid BE-FE method is 0.049 ± 0.012 at 98% source eccentricity, while the FEM is higher RDM (0.0902 ± 0.0508) at these eccentricities. On the other hand, the maximum RDM obtained from the PI-FEM is 0.1004 ± 0.0295 at 90% source eccentricity (Figure 2A).

For dipoles of radial directions, the hybrid BE-FE method significantly outperforms the BEM. The hybrid BE– FE method has a maximum RDM of 0.0639 ± 0.0189 at 98% source eccentricity and the BEM has its maximum RDM of 0.2831 ± 0.0725 at the same source eccentricity (Figure 2A). In the tangential direction, the BEM outperforms both the hybrid BE-FE method and PI-FEM at 50, 60 and 70% source eccentricities. However, its error increased and more than other approaches at 80, 90 and 98% source eccentricities. The RDM obtained from the hybrid BE-FE method is 0.049 ± 0.012 at 98% source eccentricity, while the BEM has a maximum RDM 0.1388 ± 0.0669 at 80% source eccentricities (Figure 3A).

With regard to the MAG (Figures 2B, 3B), the hybrid BE-FE method outperforms the PI-FEM and BEM. In the radial direction, the hybrid BE–FE has a maximum MAG of 1.0709 ± 0.0298 at 98% source eccentricity. While the PI-FEM and BEM have maximum MAG error of 1.1856 ± 0.2946 and 0.8178 ± 0.0273 at 90 and 98% source eccentricity, respectively (Figure 3B). In the tangential direction, the worst result of MAG from the hybrid BE-FE method is 1.0203 ± 0.0006 at 80% source eccentricity while the PI-FEM is higher MAG (1.1979 ± 0.2284) at these eccentricities. On the other hand, the maximum MAG obtained from the BEM is 1.0423 ± 0.019 at 90% source eccentricity (Figure 3B). Also, the PI-FEM leads to the largest MAG variance with *p*-value < 0.05 at all source eccentricities, as shown in Table 2.

### 4.2 Example II: anisotropic piece-wise homogenous three-layer spherical head model

For modeling anisotropicity in the EEG forward problem, the hybrid BE–FE method offers an alternative solution. So to assess the performance of the hybrid BE-FE method for the anisotropic three-layer spherical head model, we compared its performance with that of PI-FEM. The radius and conductivity of each layer were the same as those in Table 1, but the conductivity of the skull was anisotropic with an optimized anisotropy ratio 0.0093: 0.015 (Dannhauer et al., 2011). It is noteworthy that in this model, the brain (containing dipoles) is modeled by using the BEM, while other layers are modeled by using the FEM.

**Figure 4:**
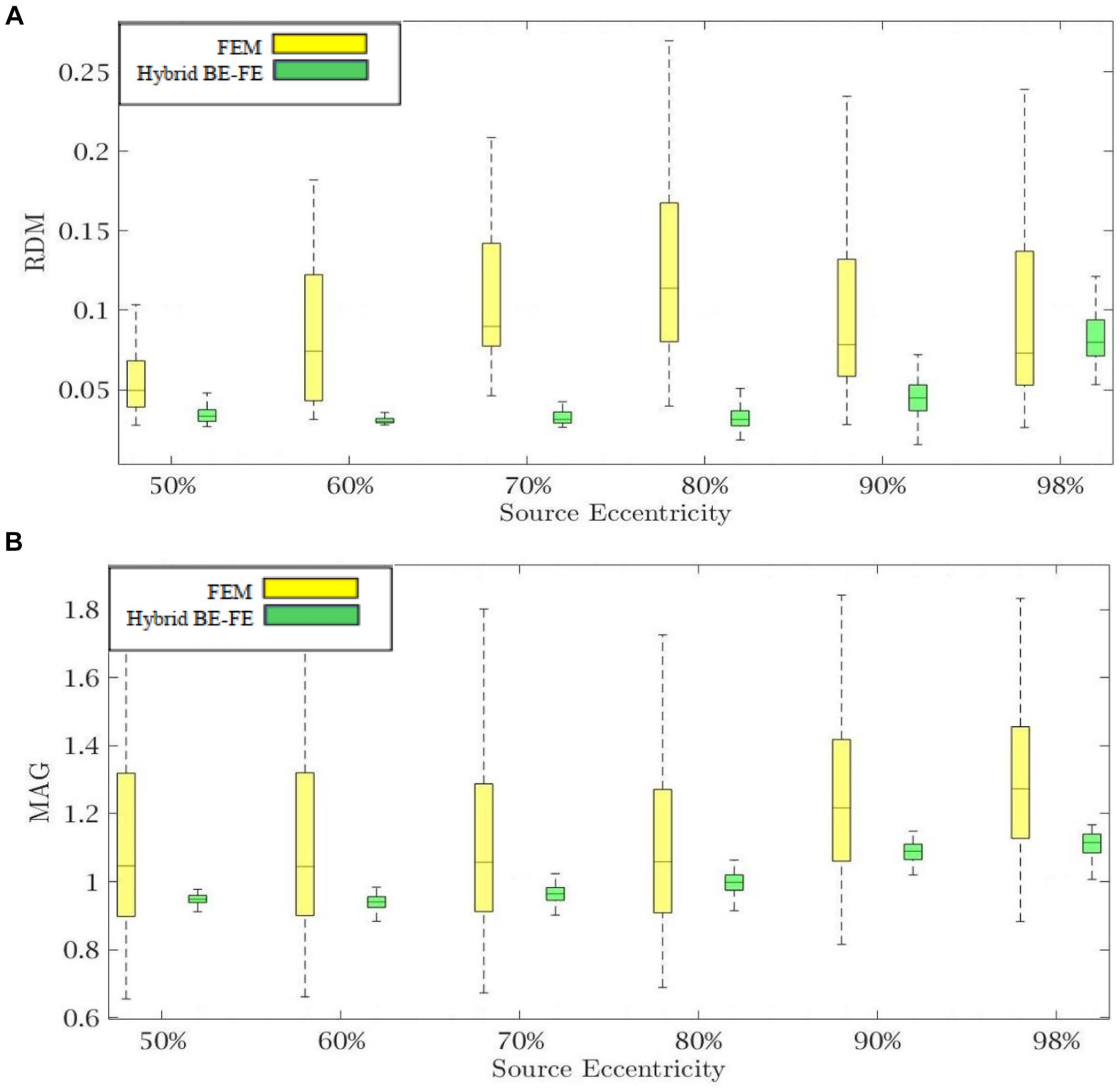
Example II: anisotropic piece-wise homogenous three-layer spherical head model for radial dipole orientation (z-axis), **(A)** RDM and **(B)** MAG boxplots of PI-FEM and hybrid BE-FE methods at six different source eccentricities.

**Figure 5:**
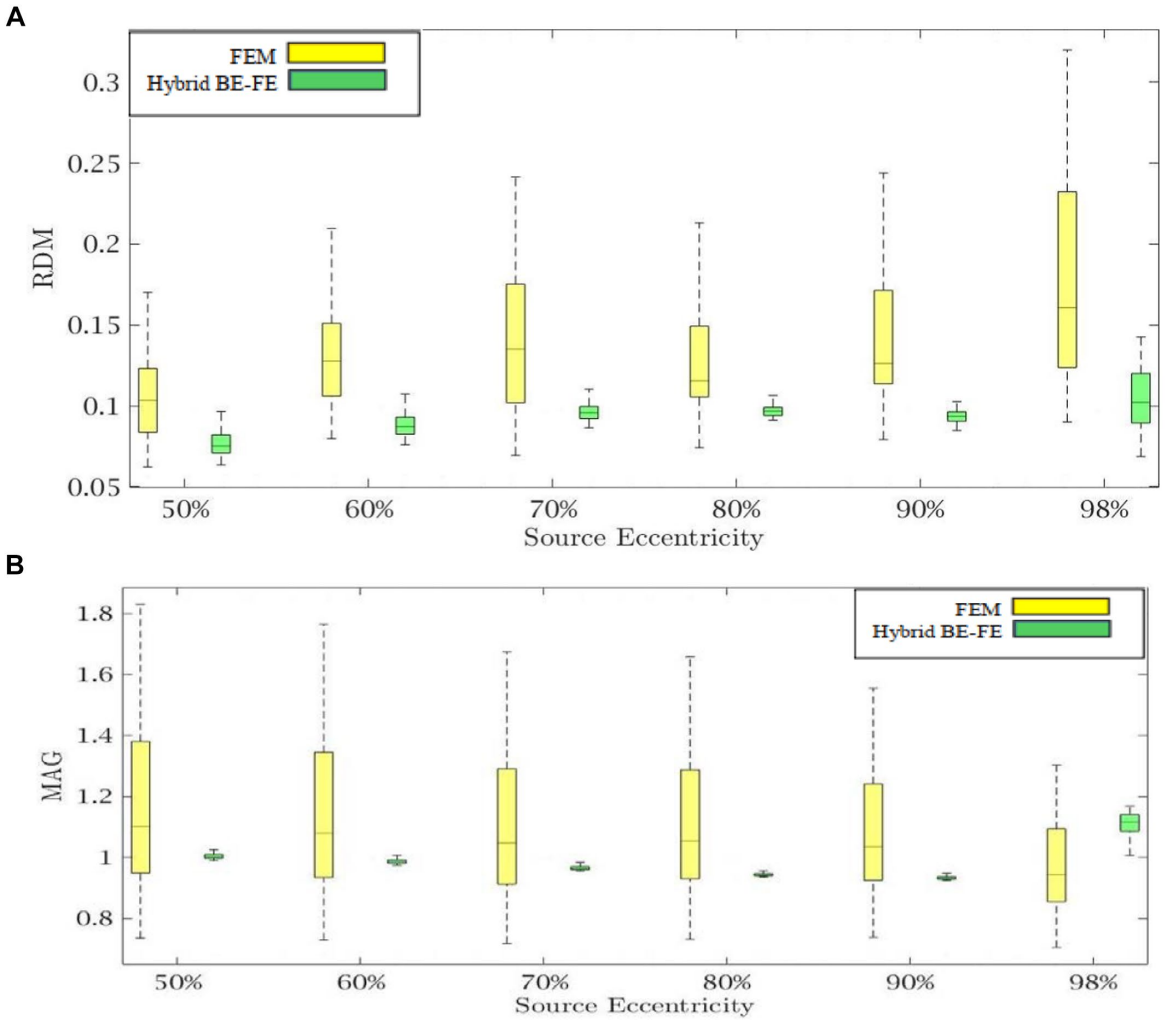
Example II: anisotropic piece-wise homogenous three-layer spherical head model for tangential dipole orientation (x-axis) **(A)** RDM and **(B)** MAG boxplots of PI-FEM and hybrid BE-FE methods at six different source eccentricities.

Figures 4, 5 show the resulting RDM and MAG for various dipole eccentricities when dipoles are radial and tangential, respectively. Also, the mean and standard deviation of RDM and MAG, and *p*-values of the Wilcoxon signed-rank test are reported in Table 3. It should be noted that some datasets did not pass the Gaussian test (*p*-value < 0.05). For this reason, we used Wilcoxon signed-rank test to calculate *p*-value. The significant differences are shown as gray in Table 3. The numbers of nodes, tetrahedral volume elements and triangular surface elements of volume mesh are the same as the previous simulation in Section 4.1.

**Table 3:** Example II: anisotropic piece-wise homogeneous three-layer spherical head model, mean ± std. and *P*-value of Wilcoxon signed-rank test of 50 realizations of RDM and MAG obtained from PI-FEM and hybrid BE-FE method for dipoles of six different source eccentricities and both radial and tangential directions.

		Radial direction (z-axis)						Tangential direction (x-axis)					
	Source eccentricity	50%	60%	70%	80%	90%	98%	50%	60%	70%	80%	90%	98%
RDM	PI- FEM	0.0562 ± 0.0206	0.0843 ± 0.0422	0.1059 ± 0.0395	0.126 ± 0.0548	0.096 ± 0.0502	0.0917 ± 0.0501	0.105 ± 0.0265	0.1312 ± 0.0314	0.1411 ± 0.0426	0.1266 ± 0.0306	0.1399 ± 0.0376	0.1762 ± 0.0594
	Hybrid BE-FE	0.0356 ± 0.0084	0.0323 ± 0.0061	0.0344 ± 0.0087	0.0322 ± 0.0075	0.0437 ± 0.014	0.08326 ± 0.0178	0.0785 ± 0.0126	0.0901 ± 0.012	0.098 ± 0.0098	0.0979 ± 0.006	0.094 ± 0.0051	0.1158 ± 0.0474
	*P*-value	1.1913 × 10^−7^	8.0311 × 10^−10^	9.0681 × 10^−10^	7.5569 × 10^−10^	1.3025 × 10^−9^	0.6887	0.237	1.0717 × 10^−7^	2.0113 × 10^−7^	2.742 × 10^−7^	7.5824 × 10^−9^	1.0872 × 10^−9^
MAG	PI-FEM	1.1072 ± 0.273	1.1035 ± 0.2646	1.105 ± 0.2517	1.0996 ± 0.2326	1.2453 ± 0.2309	1.2934 ± 0.2143	1.1564 ± 0.2656	1.1317 ± 0.2517	1.0951 ± 0.233	1.0993 ± 0.2143	1.0745 ± 0.188	0.9709 ± 0.1446
	Hybrid BE-FE	0.9474 ± 0.016	0.9372 ± 0.0255	0.9632 ± 0.0289	0.9931 ± 0.0392	1.0801 ± 0.0442	1.0925 ± 0.0722	1.0072 ± 0.0168	0.9901 ± 0.0159	0.9683 ± 0.0143	0.9454 ± 0.0115	0.9333 ± 0.0087	1.0925 ± 0.0722
	*P*-value	0.0007	0.0002	0.0014	0.0104	4.348 × 10^−5^	2.4737 × 10^−7^	0.0006	0.0026	0.0169	0.0088	0.0219	0.1333

The significant results are shown as gray.

Comparing the hybrid BE-FE method and PI-FEM with regard to the RDM (Figures 4A, 5A) shows the hybrid BE-FE method outperforms the PI-FEM in both directions, especially in the radial direction (*p*-value < 0.05) (Figure 4A). For radial dipoles, the maximum RDM obtained from the hybrid BE-FE method is 0.08326 ± 0.0178 at 98% source eccentricity, while the PI-FEM is higher RDM (0.0917 ± 0.0501) at this eccentricity. While the PI-FEM has a maximum RDM error of 0.126 ± 0.0548 at 80% source eccentricity (Figure 4A). For tangential dipoles, the PI-FEM has a maximum RDM of 0.1762 ± 0.0594 at 98% source eccentricity while the hybrid BE-FE method leads to a maximum RDM of 0.1158 ± 0.0474 at the same source eccentricity. The variance of RDM obtained from the PI-FEM is much greater than that of the hybrid BE-FE method, with *p*-value < 0.05 for all source eccentricities.

The results of MAG (Figure 4B, 5B) clearly show that the hybrid BE-FE method outperforms the PI-FEM for dipoles of both directions. The variance of MAG obtained from the PI-FEM is much greater than that of the hybrid BE-FE method with *p*-value < 0.05 for all source eccentricities.

### 4.3 Example III: anisotropic piece-wise homogenous four-layer spherical model

We simulate the anisotropic piece-wise homogenous four-layer spherical head model to assess the performance of the hybrid BE-FE method compared with the PI-FEM when a fourth layer (CSF) was considered.

Figure 1C indicates the piece-wise homogeneous four-layer spherical head model. The radius and conductivity interval of these four layers are indicated in Table 4. In this simulation, the PI-FEM mesh has 14,350 nodes and 81,942 tetrahedral volume elements, and the hybrid BE-FE mesh has 7,248 nodes, 33,855 tetrahedral volume elements and 3,550 triangular surface elements.

**Table 4:** Example III: parameters of the concentric four-layer spherical head model (Wagner et al., 2016; Vorwerk et al., 2019a).

Tissue	Brain	CSF	Skull	Scalp
Outer shell radius (cm)	7.6	8.0	8.6	9.2
Conductivity interval (S/m)	0.2200–0.6700	1.7696–1.8104	0.0016–0.0330	0.2800–0.8700
Optimized anisotropy ratio	-	-	0.0093: 0.015	-

The conductivity values, radius and optimized anisotropy ratio are based on Vorwerk et al. (2019a), Wagner et al. (2016), and Dannhauer et al. (2011), respectively.

**Figure 6:**
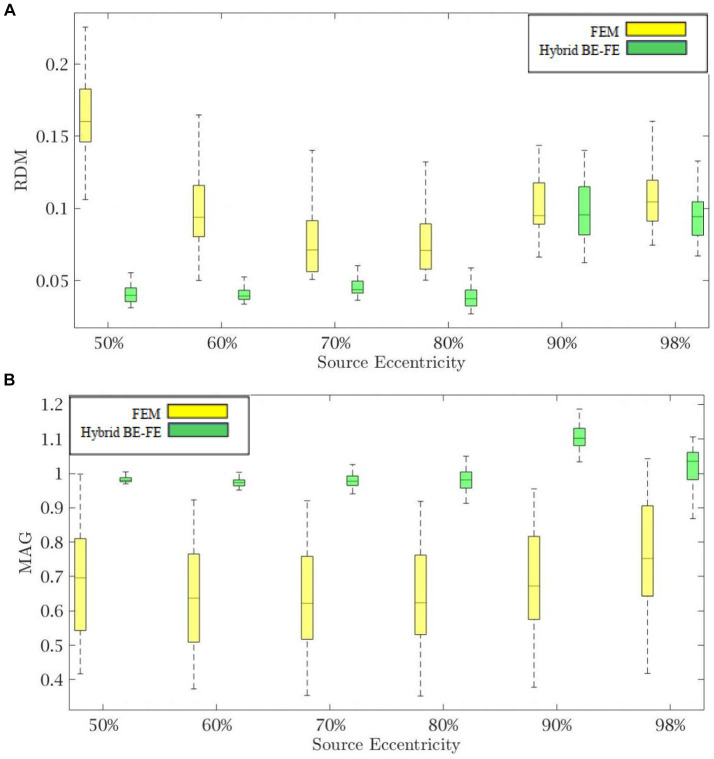
Example III: anisotropic piece-wise homogenous four-layer spherical head model for radial dipole orientation (z-axis) **(A)** RDM and **(B)** MAG boxplots of PI-FEM and hybrid BE-FE methods at six different source eccentricities.

**Figure 7:**
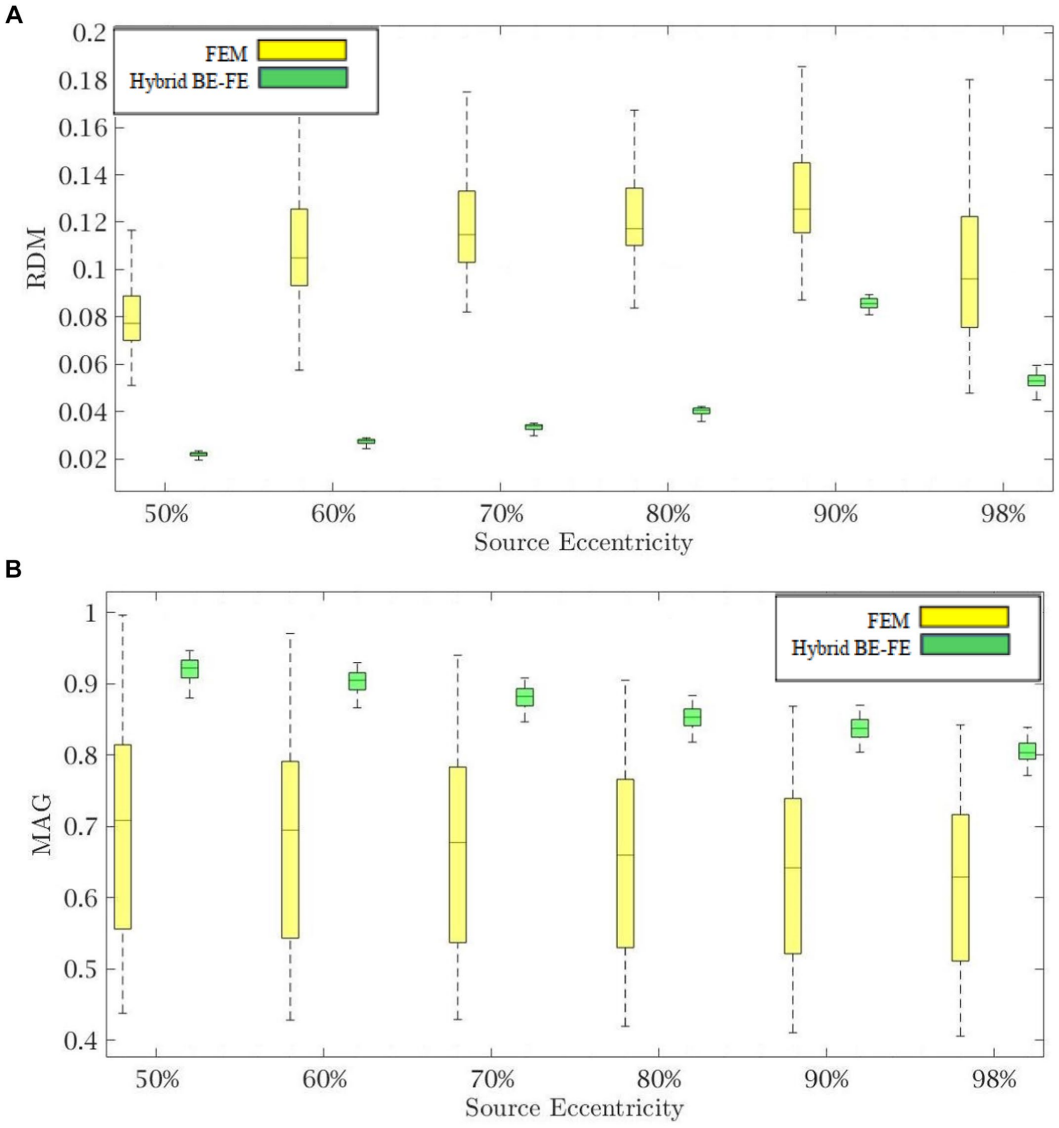
Example III: anisotropic piece-wise homogenous four-layer spherical head model for tangential dipole orientation (x-axis). **(A)** RDM and **(B)** MAG boxplots of PI-FEM and hybrid BE-FE methods at six different source eccentricities.

Figures 6, 7 show boxplots of RDM and MAG of the PI-FEM and the hybrid BE-FE method for anisotropic and piece-wise homogeneous four-layer spherical head model (Figure 1C) versus different eccentricities of the dipole for radial and tangential dipoles, respectively. For each model, 50 realizations were simulated by randomly chosen conductivities from intervals shown in Table 4. Also, the mean and standard deviation of RDM and MAG and *p*-values of Wilcoxon signed-rank tests are reported in Table 5. It should be noted that some datasets did not the pass Gaussian test (*p*-value < 0.05). For this reason, we used the Wilcoxon signed-rank test to calculate *p*-value. The significant results in this table are shown as gray.

**Table 5:** Example III: anisotropic piece-wise homogeneous four-layer spherical head model, mean ± std. and *p*-value of Wilcoxon signed-rank test of 50 realizations of RDM and MAG obtained from PI-FE and hybrid BE-FE methods for dipoles of six different source eccentricities and both radial and tangential directions.

		Radial direction (z-axis)						Tangential direction (x-axis)					
	Source eccentricity	50%	60%	70%	80%	90%	98%	50%	60%	70%	80%	90%	98%
RDM	PI- FEM	0.1641 ± 0.027	0.0992 ± 0.0281	0.0773 ± 0.0243	0.0782 ± 0.0253	0.1041 ± 0.0244	0.1072 ± 0.0215	0.0801 ± 0.0147	0.1092 ± 0.0252	0.0901 ± 0.0225	0.0941 ± 0.0218	0.1031 ± 0.025	0.1019 ± 0.0338
	Hybrid BE-FE	0.043 ± 0.0115	0.0436 ± 0.0114	0.0483 ± 0.0122	0.0385 ± 0.008	0.103 ± 0.0283	0.0984 ± 0.0256	0.0216 ± 0.0016	0.0269 ± 0.0021	0.0329 ± 0.0028	0.0395 ± 0.0031	0.0848 ± 0.0068	0.0526 ± 0.0045
	*P*-value	7.5569 × 10^−10^	0.5592	1.1713 × 10^−6^	5.3259 × 10^−6^	0.9807	0.0733	1.1962 × 10^−8^	0.023	0.0033	0.0295	0.7611	0.8963
MAG	PI- FEM	0.6797 ± 0.1572	0.6324 ± 0.1476	0.6289 ± 0.1472	0.6375 ± 0.146	0.6843 ± 0.1496	0.7623 ± 0.162	0.6903 ± 0.1489	0.6733 ± 0.1426	0.6614 ± 0.1384	0.6473 ± 0.1329	0.6306 ± 0.1248	0.615 ± 0.1179
	Hybrid BE-FE	0.9828 ± 0.0097	0.9744 ± 0.0143	0.9809 ± 0.0226	0.9783 ± 0.0355	1.1064 ± 0.0373	1.0133 ± 0.0688	0.913 ± 0.0318	0.8965 ± 0.0303	0.8743 ± 0.0287	0.8469 ± 0.0269	0.8323 ± 0.0259	0.8002 ± 0.0231
	*P*-value	7.5569 × 10^−10^	7.5569 × 10^−10^	7.5569 × 10^−10^	7.5569 × 10^−10^	7.5569 × 10^−10^	1.7569 × 10^−9^	7.5569 × 10^−10^	7.5569 × 10^−10^	7.5569 × 10^−10^	7.5569 × 10^−10^	7.5569 × 10^−10^	7.5569 × 10^−10^

The significant results are shown as gray.

As shown in Figures 6A, 7A, with regard to the RDM, the hybrid BE-FE method is more accurate than PI-FEM for both dipole directions. In fact, the mean RDM obtained from the hybrid BE-FE method is significantly smaller (*p*-value < 0.05) than that of PI-FEM for both dipole directions and at most of the eccentricities. The maximum RDM obtained from the PI-FEM is 0.1641 ± 0.027 at 50% source eccentricity at the radial direction, whereas for the hybrid BE-FE method, this value at this source eccentricity is 0.043 ± 0.0115. On the other hand, at the tangential direction (Figure 7A), the mean RDM obtained from the PI-FEM has a maximum of 0.1092 ± 0.0252 at 60% source eccentricity, while the hybrid BE-FE method has a maximum RDM of 0.0848 ± 0.0068 at 90% source eccentricity. Also, with regard to the MAG (Figures 6B, 7B), the influence of considering the CSF layer to the spherical head model is apparent. As shown in Figures 6B, 7B, the mean MAG obtained from the PI-FEM is highly decreased by considering the fourth layer. On the other hand, the MAG error obtained from the hybrid BE-FE method is significantly much better than PI-FEM (*p*-value < 0.05) in both directions. For radial dipoles, the best results of MAG for the PI-FEM and the hybrid BE- FE method are 0.7623 ± 0.162 at 98% source eccentricity and 1.0133 ± 0.0688 at the same source eccentricity. On the other hand, for tangential dipoles, the mean MAG obtained from the hybrid BE-FE method is significantly better (*p*-value < 0.05) than that of PI-FEM at all eccentricities. Also, the variance of MAG obtained from the PI- FEM is much bigger than that of the hybrid BE-FE method, which implies the hybrid BE-FE method to be more precise than PI-FEM for tangential dipoles.

### 4.4 Example IV: anisotropic piece-wise homogenous four-layer realistic head model

**Figure 8:**
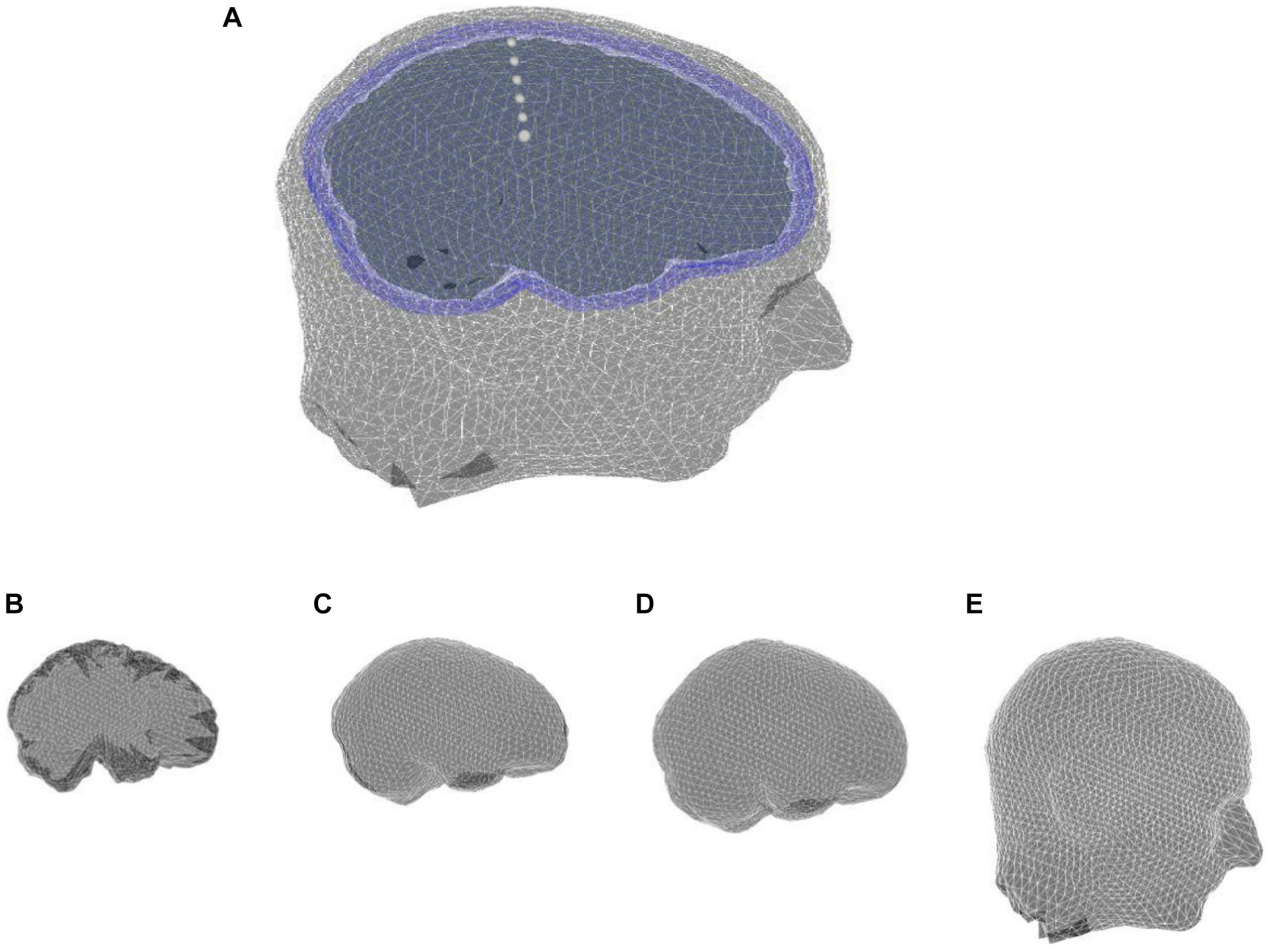
**(A)** MRI-based realistic head model with four layers: **(B)** brain, **(C)** CSF, **(D)** skull, and **(E)** scalp.

Although spherical head models (and associated analytical solutions) are fundamental for reliable assessment of any proposed forward solution strategy, it is of fundamental importance to validate the applicability and performance of the technique proposed here on realistic MRI-obtained head models (Figure 8). These models allow for an individual-based head model to be used in solving the forward problem and result in more precise source localization.

The single-subject anatomical MRI used in this study, has been available in Fieldtrip. It is scaled based on one of the templates from the Montreal Neurological Institute (MNI) (Holmes et al., 1998; Oostenveld et al., 2011). The values of conductivity of brain, CSF and scalp layers are considered 0.33, 1.79 and 0.43 S/m, respectively (Vorwerk et al., 2019a). Although the three-layer heterogeneous skull model is more accurate than a single-layer homogenous and anisotropic the skull, we used the simplified model of the skull as a single-layer homogenous and anisotropic profile obtained by automatic segmentation of FieldTrip software (Oostenveld et al., 2011). However, our method is able to model a complex profile of the skull when it is available. We use optimized anisotropy ratio 0093/0.015 S/m for skull in this study (Dannhauer et al., 2011).

In the realistic head model, the PI-FEM mesh has 221,779 nodes and 1,380,065 tetrahedral volume elements, and the hybrid BE-FE mesh has 108,623 nodes, 655,756 tetrahedral volume elements and 7,004 triangular surface elements. Since the analytical solution is unavailable in this case, the PI-FEM served as the reference method.

The forward problem was solved by using the PI-FEM on a refined model having 10,850,052 tetrahedrons and 1,717,389 nodes.

**Figure 9:**
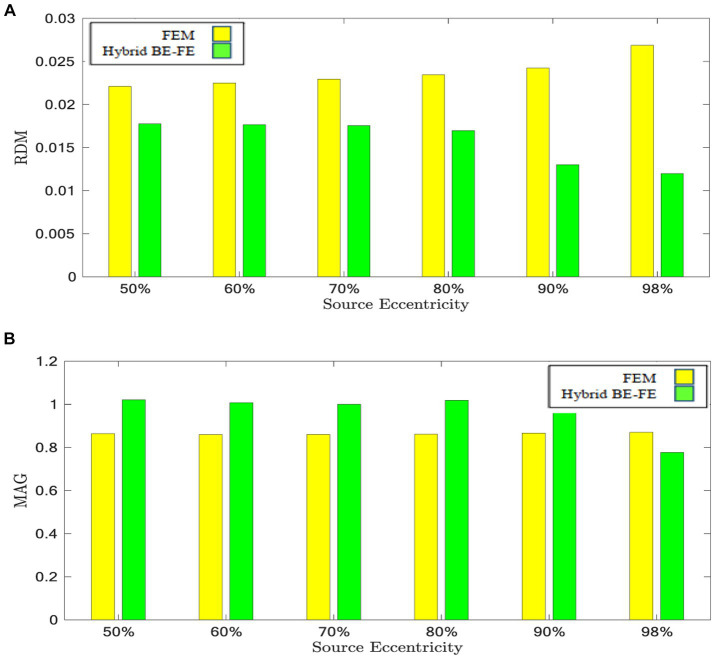
Example IV: anisotropic piece-wise homogenous four-layer realistic head model for radial dipole orientation (z-axis), **(A)** RDM and **(B)** MAG boxplots of PI-FEM and hybrid BE-FE methods at six different source eccentricities.

**Figure 10:**
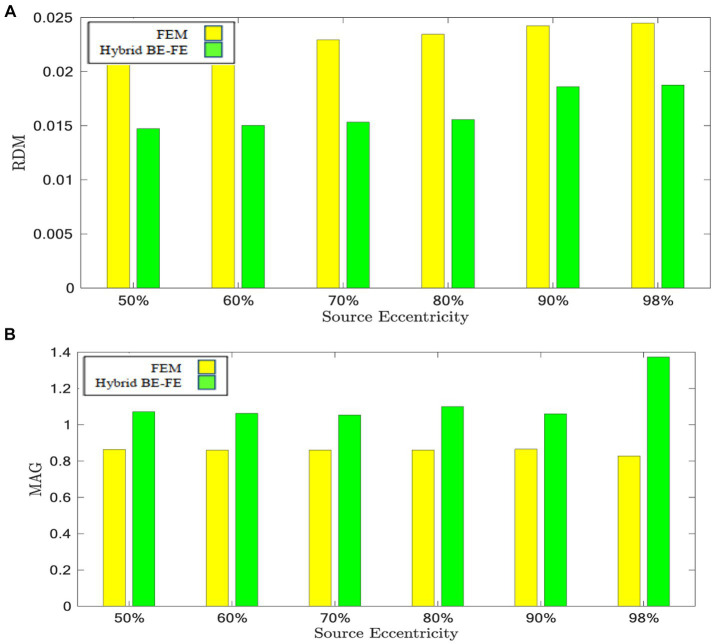
Example IV: anisotropic piece-wise homogenous four-layer realistic head model for tangential dipole orientation (x-axis). **(A)** RDM and **(B)** MAG boxplots of PI-FEM and hybrid BE-FE methods at six different source eccentricities.

The results of this benchmarking can be seen in Figures 9, 10 for radial and tangential dipoles, respectively, where a dipolar source was moved from 50 to 98% of the source eccentricity from the center to the surface of the brain, as shown in Figure 8. The RDM and MAG obtained from the hybrid BE-FE method, and PI-FEM presented in this section have been computed with respect to the reference solution.

The hybrid BE-FE method outperforms the PI-FEM with regard to the RDM in both directions (Figures 9A, 10A). The highest RDM values obtained from the hybrid BE-FE method and PI-FEM, for radial dipoles, are 0.0177 at 50% source eccentricity and 0.02686 at 98% source eccentricity, respectively, and for tangential dipoles, are 0.0187 and 0.0245 respectively, both at 98% source eccentricity.

The MAG analysis (Figures 9B, 10B) showed the same results as the RDM except in 98%. The worst MAG values of the hybrid BE-FE method and PI-FEM are, respectively, 0.7771 at 98% source eccentricity and 0.8598 at 60% source eccentricity for radial dipoles, and 1.3734 and 0.8284 at 98% source eccentricity for tangential dipoles. These results may only be seen as hints since no exact solution exists that can be taken as reference.

## 5 Discussion

Although each BEM and FEM have several advantages, they have drawbacks in solving EEG forward problem. BEM cannot model complex geometry such as inhomogeneity and anisotropicity and surfaces with holes head model (de Munck et al., 2017; Rahmouni et al., 2017). On the other hand, using the FEM causes singularity in right-hand side of EEG forward Equation (1) (Zhang et al., 2014; de Munck et al., 2017). The coupling method proposed in Olivi et al. (2010) for iteratively solving the EEG forward problem has two major drawbacks. First, it is very time-consuming to achieve the relative residuals below a properly set value. Second, to solve the global system iteratively, relaxation parameters need to be set at the interfaces to ensure convergence. These relaxation parameters are set manually, and inappropriate values of these parameters would make the scheme diverge. The values of relaxation parameters are not accurate. For using the advantages of both methods, the hybrid BE–FE method offers an alternative solution. The idea behind the hybrid BE-FE method has already been proposed for solving EIT forward problem (Ghaderi Daneshmand and Jafari, 2013). In this study, we proposed reformulating the hybrid BE– FE method for solving the EEG forward problem. The hybrid BE-FE method provides an elegant solution to the practical problem of EEG forward problem of how to model heterogeneity and anisotropicity in tissues whose boundaries are known, without complex volume meshing of the whole 3D domain. It is noteworthy that in this method, the volume meshing is not eliminated, but it is limited to regions without dipoles.

Using the PI-FEM, BEM, and hybrid BE-FE method, we compared simulated results for spherical and realistic head models at six different dipole eccentricities and for radial and tangential dipoles. In this study, a layer homogenous isotropic/anisotropic skull model with optimized value was used.

Regarding RDM and MAG, the results of the isotropic inhomogeneous three-layer spherical head model showed that the hybrid BE-FE method outperforms the BEM, especially at the radial direction (see Figures 2, 3). However, with regard to RDM, the BEM performs better than the hybrid BE-FE method at 50, 60 and 70% of source eccentricities in the tangential direction, but it performs the worst at 80, 90 and 98% of source eccentricities and with regard to MAG the hybrid BE-FE method outperforms the BEM. To be noticed that the BEM cannot simulate inhomogeneous and anisotropic media, the hybrid BE-FE method can be a good alternative to be used instead of the BEM in the multi-layer medium.

In the isotropic three-layer spherical head model, the PI-FEM has the worst performance at 50, 60 and 70% of source eccentricities in both directions (see Figures 2, 3). Also, it has worse performance than the hybrid BE-FE method in all eccentricities. Nevertheless, the hybrid BE-FE method clearly performs well. On the other hand, the MAG of the PI-FEM has a large variance in both directions. While the hybrid BE-FE method has the best results in both directions.

By considering the skull as a layer homogenous anisotropic model, results showed that the hybrid BE-FE method outperforms the PI-FEM at all eccentricities in both directions (Figures 4, 5). In the radial direction, the RDM of the hybrid BE-FE method is much smaller than the PI-FEM. On the other hand, although the difference between their RDM in the tangential direction is not as much as in the radial direction, the RDM of the hybrid BE-FE method is still better than the PI-FEM (see Figures 4A, 5A). Therefore, the hybrid BE-FE method clearly performs well and has small RDM less than 0.08326 for radial direction and 0.1158 for tangential direction. While the PI-FEM has a maximum RDM of 0.126 and 0.1762 in radial and tangential direction, respectively (Table 3). On the other hand, the MAG of the PI-FEM has a large variance in both directions (see Figures 4B, 5B). While the hybrid BE-FE method has the best results in both directions.

In the next step, we compared the PI-FEM and hybrid BE-FE method when a fourth layer (CSF) was considered. With regard to the RDM, in radial direction, the hybrid BE-FE method is more accurate than the PI-FEM (see Table 5). With regard to the MAG, the hybrid BE-FE method performs clearly better than the PI-FEM. At the tangential direction, the hybrid BE-FE method outperforms the PI-FEM with regard to the RDM and MAG.

The results of the spherical head model show that the hybrid BE-FE method has higher accuracy than the PI-FEM in both directions. Also, the variance of the PI-FEM is very higher than the hybrid BE-FE method. It shows that by variation of conductivity, the performance of the hybrid BE-FE method is more stable than the PI-FEM. The comparison with the hybrid BE-FE method and PI-FEM for dipoles of different orientations and eccentricities showed that the hybrid BE-FE method leads to higher accuracy.

The result of the realistic head model shows that the hybrid BE-FE method outperforms the PI-FEM in both directions regarding RDM. Also, with regard to MAG, the hybrid BE-FE method outperforms the PI-FEM in both directions except at 98% source eccentricity. However, no exact solution exists as a reference to conclude about the realistic head model.

The results obtained from the single reference utilizing the coupling formulation of a three-dimensional domain decomposition method (3-DD) for solving the EEG forward problem yielded RDM ranging from 0.012 to 0.13 for a three-layer anisotropic spherical head model (Olivi et al., 2010). The achieved outcomes were based on dipoles oriented along the z-axis in the x, y, and z directions. Notably, the referenced study only presented results using the RDM criterion. Remarkably, our findings align with the outcomes of this study. It is important to note, however, the research employed an iterative approach, conducting their coupling method with 40 iterations.

The overall higher accuracy of the hybrid BE-FE method was expected due to theoretical considerations behind the hybrid BE-FE method since it uses each of the BEM and FEM on the domains better suited for them. It uses the BEM to model the brain layer containing dipoles to avoid the singularity problem of the FEM. On the other side, it uses the FEM to model inhomogeneous and anisotropic compartments to overcome the BEM disability in modeling inhomogeneity and anisotropicity.

There are some limitations in our study that should be addressed. First, the hybrid BE-FE method is more time consuming than the PI-FEM. For example, in the four-layer spherical head model with the same DOF, in our computer simulation study at hand, on the Microsoft Windows 10 Enterprise N, PC with Intel core i7-4510U 2.6- GHz CPU and 6-GB RAM, the total forward simulation time of the hybrid BE-FE method was three times more than the FEM. Also, the extracting mesh algorithm of the hybrid BE-FE method is more complex than FEM. There are some academic software tools that generating volume mesh for the FEM very fast and accurately. But to generate mesh for the hybrid BE-FE method, first, we need to generate volume mesh, then to extract mesh to use in the hybrid BE-FE method. Hence, it is more time consuming and complex than the FEM.

## 6 Conclusion

We presented the theory, verification, and evaluation of a hybrid BE-FE method for solving the EEG forward problem. The simulation results of spherical head models demonstrated that the hybrid BE-FE method is more accurate and precise than the PI-FEM. The error (measured by RDM and MAG) obtained from the hybrid BE-FE method indicated that it performs well for deep dipoles. However, when dipoles became close to the first conductivity jump, the error of the hybrid BE-FE method increases, but still less than the PI-FEM at the same eccentricities.

The EEG forward simulation in the realistic head model showed that the hybrid BE-FE method outperforms the PI-FEM in both directions. Overall, our simulations confirm that the hybrid BE-FE method is a promising new approach for solving heterogeneous isotropic/anisotropic EEG forward problems that can outperform the FEM.

To maintain methodological consistency, other influencing factors, including the accuracy of head layer segmentation and mesh quality, should also be considered. So, the directions of the future work can include further development of the proposed method in other properties of head layers, such as the radius of layers and different meshes. Also, we have an idea to develop a mesh generation algorithm to be used in well-known academic software packages and to be simulated faster. Moreover, we can compare the FEM and our proposed hybrid BE-FE method for a three-layer heterogeneous skull model and consider the anisotropic profile of white matter. Additionally, developing the methodology for use in some applications, such as increasing the accuracy of source localization to detect some diseases, is an attractive field of future research.

## Data availability statement

The original contributions presented in the study are included in the article/supplementary material, further inquiries can be directed to the corresponding author.

## Author contributions

ND: Conceptualization, Formal analysis, Methodology, Validation, Visualization, Writing – original draft, Writing – review & editing, Investigation. AK: Methodology, Writing – review & editing, Conceptualization, Investigation, Supervision.

## Appendix 1

TABLE A1 List of abbreviations.

**Table:**

EEG	Electroencephalography
FE	Finite element
FEM	Finite element method
PI-FEM	Partial integration- finite element method
BE	Boundary element
BEM	Boundary element method
RDM	Relative difference measure
MAG	Magnitude ratio
MRI	Magnetic resonance imaging
